# Sex- and Age-Specific Differences in the Joint Effects of Diet Quality and Physical Activity on Depressive Symptoms in Adults: A Cross-Sectional Study

**DOI:** 10.3390/nu18060915

**Published:** 2026-03-13

**Authors:** Soyoung Kim, Minseon Park

**Affiliations:** 1 Department of Family Medicine, Seoul National University Hospital, Seoul 03080, Republic of Korea; soooooy7759@gmail.com; 2 Department of Family Medicine, College of Medicine, Seoul National University, Seoul 03080, Republic of Korea

**Keywords:** healthy eating index, healthy diet, physical activity, depressive symptom, sex differences

## Abstract

**Background/Objectives:** Diet quality and physical activity are modifiable lifestyle factors linked to depressive symptoms, but their joint association—and potential variation by sex and age—remains unclear, particularly in Asian populations. This study examined the joint association of diet quality, measured by the Korean Healthy Eating Index (KHEI), and physical activity (PA) with depressive symptoms in Korean adults. **Methods:** We analyzed 17,737 adults aged ≥20 years from 2014, 2016, 2018, and 2020 KNHANES. Diet quality and PA were each dichotomized at the median to construct four lifestyle groups. Depressive symptoms were defined as Patient Health Questionnaire-9 (PHQ-9) ≥ 10. Survey-weighted multivariable logistic regression adjusted for sociodemographic factors, health-related behaviors, and major chronic diseases was performed, and stratified analyses were conducted by sex and age to explore potential variation across subgroups. **Results:** Overall, 4.6% of participants had depressive symptoms. Compared with the Low KHEI & Low PA group, the High KHEI & High PA group had substantially lower odds of depressive symptoms (aOR 0.55, 95% CI 0.42–0.73). In sex-stratified analyses, this joint association was significant only among women (aOR 0.48, 95% CI 0.35–0.66). In age-stratified analyses, significant associations were observed in adults aged 45–65 years (aOR 0.42, 95% CI 0.26–0.66) and ≥65 years (aOR 0.41, 95% CI 0.27–0.63). **Conclusions:** High diet quality combined with high physical activity was associated with markedly lower odds of depressive symptoms, particularly among women and middle-aged to older adults. These findings support integrated lifestyle strategies targeting both diet quality and physical activity, tailored to demographic and social contexts to enhance mental-health benefits across diverse populations.

## 1. Introduction

Depression has emerged as a major public-health issue across the globe, with its burden steadily increasing in both prevalence and severity [1]. South Korea is of particular concern, as national reports continue to show elevated levels of depressive symptoms and suicide rates compared with other high-income countries [2]. This pattern underscores the urgent need to identify lifestyle factors that can be targeted to improve mental-health outcomes at the population level.

A wide range of determinants contribute to the onset and progression of depressive symptoms, including biological factors, psychosocial stressors, and health-related behaviors [3]. Among these, diet quality and physical activity have consistently been highlighted as important behavioral factors linked to emotional and psychological well-being. Previous epidemiological and meta-analytic studies have shown that healthier dietary patterns and higher levels of physical activity are each inversely related to depression risk [4,5,6]. Building on this evidence, a recent NHANES-based investigation in the United States demonstrated that individuals with both higher diet quality and greater physical activity exhibited the lowest prevalence of depressive symptoms, suggesting that the joint association of these two behaviors may be more pronounced than their independent effects [7]. The biological and behavioral mechanisms underlying these associations are multifaceted. Dietary components such as omega-3 fatty acids, B vitamins, and polyphenols have been shown to modulate neuroinflammatory pathways, neurotransmitter synthesis, and hypothalamic–pituitary–adrenal axis reactivity, each of which has been implicated in depressive pathophysiology [8,9]. Physical activity, in turn, promotes neuroplasticity through increased brain-derived neurotrophic factor (BDNF) expression, reduces cortisol levels, and enhances social engagement, all of which may buffer against depression [10,11]. These pathways are not mutually exclusive, and the behavioral regularity associated with maintaining both a healthy diet and an active lifestyle may further reinforce psychological resilience through shared mechanisms.

However, evidence on the joint association of diet quality and physical activity with depressive symptoms in Asian populations remains scarce. Cultural differences in eating habits, physical activity patterns, and stress-coping mechanisms may modify the relationship between lifestyle factors and mental health, limiting the generalizability of findings from Western cohorts [12,13,14,15]. To our knowledge, no study has comprehensively examined the joint association of diet quality and physical activity with depressive symptoms using nationally representative data from an Asian country. Beyond geographic generalizability, the analytical approach of examining joint lifestyle categories—rather than modeling diet and physical activity as independent predictors—offers distinct advantages. This approach reflects the co-occurrence of these behaviors in real-world settings and allows identification of the lifestyle profile associated with the greatest mental health benefit, which is more directly translatable to public health recommendations. Furthermore, the use of the Korean Healthy Eating Index (KHEI), an index developed specifically to reflect Korean dietary guidelines and food culture, enables a culturally grounded assessment of diet quality that is not adequately captured by Western dietary indices such as the Healthy Eating Index (HEI) or Mediterranean Diet Score (MDS). Sex- and age-stratified analyses add a further dimension by identifying subgroups for whom lifestyle-based interventions may be particularly relevant.

To address this evidence gap, the present study examined the joint association of diet quality and physical activity with depressive symptoms among adults using nationally representative data from Korea, contributing context-specific evidence from an Asian population. We hypothesized that individuals with both high diet quality and high physical activity would have lower odds of depressive symptoms compared with those with both low diet quality and low physical activity, and that these associations would vary by sex and age, with the aim of informing the development of tailored mental-health promotion strategies.

## 2. Materials and Methods

### 2.1. Study Participants

We used data from the Korea National Health and Nutrition Examination Survey (KNHANES), a nationwide, cross-sectional survey of the non-institutionalized Korean population that employs a multistage, stratified cluster sampling design. Data were pooled from four survey cycles (2014, 2016, 2018, and 2020), in which KHEI and PHQ-9 data were obtained using comparable protocols. Further methodological details are available from the official website (https://knhanes.kdca.go.kr, accessed on 11 March 2026), where the survey data and documentation are publicly accessible for research use.

**Figure 1:**
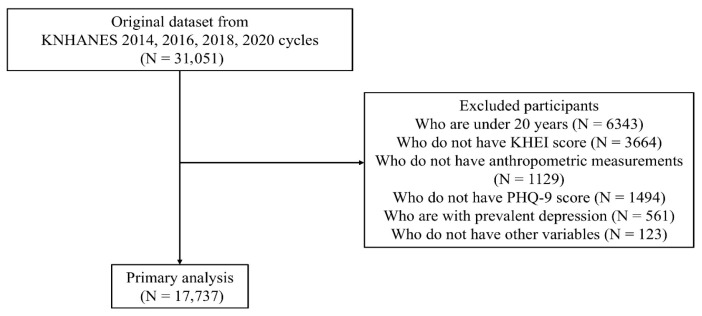
Flow Diagram of Participant Selection. The flow diagram presents the inclusion and exclusion process used to derive the analytic sample from the 2014, 2016, 2018, and 2020 cycles of the KNHANES. Adults aged ≥20 years were eligible for inclusion. Participants were sequentially excluded if they lacked KHEI scores, anthropometric measurements, PHQ-9 data, had prevalent depression, or were missing other required variables. The final analytic sample comprised 17,737 adults.

We restricted the sample to adults aged ≥20 years. Participants were then excluded sequentially if they did not have KHEI scores, lacked anthropometric measurements (height or weight), did not have PHQ-9 data, had prevalent depression (defined as a self-reported current diagnosis of depression) to minimize potential reverse causality and treatment-related confounding of PHQ-9 scores, or were missing other covariates required for the multivariable analyses. After applying these criteria, a total of 17,737 participants were included in the final analytic sample. The detailed process of participant selection is illustrated in Figure 1.

The KNHANES protocols were approved by the Institutional Review Board of the Korea Centers for Disease Control and Prevention as follows: the 2014 survey (IRB No. 2013-12EXP-03-5C), the 2016 and 2018 surveys (IRB No. 2018-01-03-P-A), and the 2020 survey (IRB No. 2018-01-03-2C-A). Informed consent was obtained from all participants, and all data were anonymized before analysis.

### 2.2. Assessment of Diet Quality

Dietary intake information was obtained from the nutrition survey of KNHANES, which includes a one-day 24 h dietary recall and a semi-quantitative food frequency questionnaire (FFQ), both administered by trained dietitians. The 24 h recall data were used to estimate daily energy and nutrient intake based on the Korean Food Composition Table. The FFQ was designed to capture participants’ usual dietary patterns over the previous year [16].

Diet quality was evaluated using the Korean Healthy Eating Index (KHEI), a validated measure developed to assess adherence to national dietary guidelines. The KHEI is a composite score ranging from 0 to 100 and consists of 14 components across three domains. The adequacy domain includes having breakfast, mixed grain intake, total fruit intake, fresh fruit intake, total vegetable intake, vegetable intake excluding Kimchi and pickled vegetables, meat–fish–egg–bean intake, and milk and milk product intake. The moderation domain comprises the percentage of energy from saturated fatty acids, sodium intake, and the percentage of energy from sweets and beverages. The balance of energy intake domain evaluates the percentage of energy from carbohydrates, the percentage of energy from fat, and total energy intake. Higher scores indicate better overall diet quality and closer adherence to recommended dietary patterns [17].

For analytical purposes, participants were categorized into “low” (15.01–64.07) and “high” (64.07–99.82) KHEI groups based on the median value of the total score in the analytic sample as the cutoff, which was similar to previously reported average KHEI values (63.20) in Korean adults [17].

### 2.3. Assessment of Physical Activity

Physical activity (PA) was assessed via a self-reported questionnaire, which collected information on the frequency and duration of vigorous, moderate, and walking activities during a typical week. For each activity domain, total weekly minutes were calculated by multiplying the number of days per week by the average duration per day. Based on the standard metabolic equivalent of task (MET) values, energy expenditure for each domain was computed using the formula kcal/min = 3.5 × MET × body weight (kg)/200, where MET values of 8.0, 4.0, and 3.3 were assigned to vigorous, moderate, and walking activities, respectively. The daily physical activity energy expenditure for each activity was calculated by dividing the total weekly energy expenditure by seven and summing across all activity domains [18,19].

To classify participants by physical activity level, PA was dichotomized into “low” (0–100.48 kcal/day) and “high” (100.48–4160.16 kcal/day) PA groups using the median value of the analytic sample as the cutoff, which approximates the activity level associated with protective effects against depression in previous meta-analytic studies [20].

### 2.4. Assessment of Depressive Symptoms

Depressive symptoms were assessed using the Korean version of the Patient Health Questionnaire-9 (PHQ-9). The PHQ-9 consists of nine items corresponding to the diagnostic criteria for major depressive disorder in the Diagnostic and Statistical Manual of Mental Disorders, Fourth Edition (DSM-IV). Each item asks how often the participant experienced specific depressive symptoms during the past two weeks, with response options ranging from 0 (“not at all”) to 3 (“nearly every day”). The total score thus ranges from 0 to 27, with higher scores indicating greater depressive symptom severity [21].

In accordance with established guidelines and previous studies using KNHANES data [22,23], participants with a PHQ-9 score of 10 or higher were classified as having depressive symptoms, representing moderate to severe symptom levels. Those with scores below 10 were classified as without depressive symptoms.

The internal consistency of the PHQ-9 in the Korean population has been well validated, with Cronbach’s α values typically around 0.80, indicating high reliability [24]. In the present analytic sample, Cronbach’s α was 0.78, confirming adequate internal consistency.

### 2.5. Other Variables

Sociodemographic and health-related variables were selected based on previous literature and their potential associations with depressive symptoms [22,23].

Age, sex, height, and weight were collected as part of the health examination, and body mass index (BMI) was calculated as weight in kilograms divided by height in meters squared (kg/m^2^).

Socioeconomic variables included household income, educational level, marital status, living status, and eating type. Education level was categorized into three levels: less than high school, high school graduate, and beyond high school. Household income was classified into tertiles (low, middle, and high) based on the equivalized income distribution. Marital status was grouped as married or unmarried, including those who were never married, divorced, or widowed. Living status was categorized as living alone or living with others, and eating type was classified as eating together, eating partially together, or eating alone.

Health-related behavior variables included smoking status and alcohol consumption. Smoking was categorized as never, former, or current smoker. Alcohol consumption was classified into three groups according to the KNHANES definition: non-drinker, non–high-risk drinker, and high-risk drinker (≥7 drinks per occasion and ≥2 times per week for men; ≥5 drinks per occasion and ≥2 times per week for women).

Clinical variables included self-reported physician-diagnosed chronic diseases and medication use. Participants were asked whether they had ever been diagnosed with cardiovascular or cerebrovascular disease (CCVD), cancer, or were currently taking medications for hypertension, diabetes mellitus (DM), or dyslipidemia.

### 2.6. Statistical Analysis

Continuous variables were summarized as mean ± standard deviation (SD), and categorical variables as numbers and percentages. Differences in participant characteristics according to depressive symptom status were examined using Student’s *t*-test for continuous variables and the χ^2^ test for categorical variables.

Diet quality, represented by the KHEI, and PA were each divided into “low” and “high” groups using the median values of the analytic sample as cutoffs. Participants were then classified into four combined categories: Low KHEI & Low PA, High KHEI & Low PA, Low KHEI & High PA, and High KHEI & High PA. The Low KHEI & Low PA group served as the reference category. To assess the robustness of this the associations, sensitivity analyses using KHEI and PA as continuous variables and as quartiles were additionally conducted (Supplementary Table S3).

Survey-weighted binary logistic regression analysis was used to estimate the associations between diet quality, physical activity, and depressive symptoms, accounting for the complex sampling design of KNHANES including stratification, clustering, and sampling weights. As data were pooled across four survey cycles, sampling weights were divided by four to account for the multi-cycle pooling, following recommended procedures for combined KNHANES analyses. All models were fully adjusted for sociodemographic, lifestyle, and clinical variables, including age, sex, BMI, household income, educational level, marital status, living status, eating type, smoking status, alcohol consumption, CCVD, cancer, and medication use for hypertension, DM, or dyslipidemia. In addition, subgroup analyses were conducted by sex (men, women), age (20–45, 45–65, and ≥65 years), BMI (<18.5, 18.5–23, 23–25, and ≥25 kg/m^2^), living status, and eating type.

All statistical analyses were performed using R software (version 4.3.2; R Foundation for Statistical Computing, Vienna, Austria). Survey-weighted regression analyses were performed using the svyglm function from the survey package in R. All statistical tests were two-sided, and *p* values < 0.05 were considered statistically significant.

## 3. Results

### 3.1. Baseline Characteristics of Study Participants

A total of 17,737 adults were included in the analytic sample. The mean age was 51.6 years, and 57.8% were women. Participants were categorized into four lifestyle groups: Low KHEI & Low PA (24.0%), High KHEI & Low PA (22.0%), Low KHEI & High PA (25.4%), and High KHEI & High PA (28.6%) (Table 1).

Several sociodemographic and behavioral characteristics differed significantly across lifestyle groups. The High KHEI & Low PA group included a higher proportion of older adults and women, whereas the Low KHEI & High PA group comprised more younger adults and men (all *p* < 0.001). Higher household income and higher educational level were more common in the High KHEI & High PA group, while the Low KHEI & Low PA group had greater proportions of individuals living alone and eating alone (all *p* < 0.001). Health-related behaviors also varied across groups: current smoking and high-risk drinking were more frequent in the Low KHEI & High PA group and less common in the High KHEI & Low PA group (all *p* < 0.001). Chronic conditions significantly differ across lifestyle groups. CCVD was most prevalent in the Low KHEI & Low PA group, whereas cancer, hypertension, DM, and dyslipidemia were more common in the High KHEI & High PA group (all *p* < 0.001).

Among the 17,737 participants, 822 (4.6%) had depressive symptoms. Of these, 570 (69.3%) had moderate symptoms (PHQ-9 10–14), 179 (21.8%) had moderately severe symptoms (PHQ-9 15–19), and 73 (8.9%) had severe symptoms (PHQ-9 20–27). Among those without depressive symptoms, 14,530 (82.0%) had minimal symptoms (PHQ-9 0–4) and 2385 (13.5%) had mild symptoms (PHQ-9 5–9). As shown in Table 2, participants with depressive symptoms were more likely to be older, women, to have lower household income and lower educational level, and to have a higher prevalence of living alone and eating alone (all *p* < 0.001). Unhealthy behaviors were also more common in this group, including higher proportions of current smoking and high-risk drinking. Clinically, depressive symptoms were associated with higher prevalence of CCVD, cancer, hypertension, DM, and dyslipidemia.

### 3.2. Association Between Diet Quality, Physical Activity and Depressive Symptoms

Table 2 shows that diet quality declined progressively with increasing severity of depressive symptoms, with mean KHEI scores of 63.93 ± 13.18 in the minimal, 61.64 ± 13.44 in the mild, 60.27 ± 13.94 in the moderate, 57.80 ± 13.66 in the moderately severe, and 54.82 ± 13.62 in the severe group (*p* < 0.001). Lower scores were observed across most adequacy components, including breakfast, fruits, vegetables, and meat–fish–egg–and–beans. Physical activity also showed a similar gradient, declining progressively from 188.39 ± 252.46 kcal/day in the minimal group to 130.52 ± 241.49 kcal/day in the severe group (*p* < 0.001). Consistently, the distribution of lifestyle groups indicated that the Low KHEI & Low PA group was most prevalent among those with severe depressive symptoms (50.7%), whereas the High KHEI & High PA group was most common among those with no depressive symptoms (30.4%) (*p* < 0.001).

**Figure 2:**
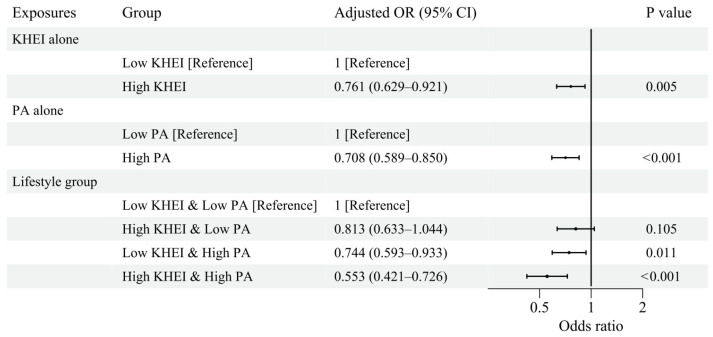
Associations of Diet Quality, Physical Activity, and Lifestyle Groups with Depressive Symptoms. Survey-weighted fully adjusted odds ratios (aORs) and 95% confidence intervals for depressive symptoms according to diet quality (KHEI), physical activity (PA), and lifestyle groups. Models were adjusted for age, sex, BMI, household income, educational level, marital status, living status, eating type, smoking status, alcohol consumption, CCVD, cancer, and medication use for hypertension, DM, or dyslipidemia. The Low KHEI & Low PA group served as the reference category.

Figure 2 presents the associations between diet quality, physical activity, and depressive symptoms in the survey-weighted fully adjusted models. Higher diet quality was independently associated with lower odds of depressive symptoms, with participants in the high KHEI group showing a 24% reduction in odds compared with those in the low KHEI group (aOR 0.761; 95% CI, 0.629–0.921). Higher physical activity was also associated with lower odds of depressive symptoms (aOR 0.708; 95% CI, 0.589–0.850). When diet quality and physical activity were examined jointly, a clear gradient was observed across lifestyle groups. Compared with the Low KHEI & Low PA group, the High KHEI & Low PA group did not show a statistically significant association (aOR 0.813; 95% CI, 0.633–1.044; *p* = 0.105), while the Low KHEI & High PA group showed significantly lower odds of depressive symptoms (aOR 0.744; 95% CI, 0.593–0.933). The greatest reduction was observed in the High KHEI & High PA group, which demonstrated approximately 45% lower odds of depressive symptoms (aOR 0.553; 95% CI, 0.421–0.726).

### 3.3. Subgroup Analyses of Association Between Diet Quality, Physical Activity and Depressive Symptoms

**Figure 3:**
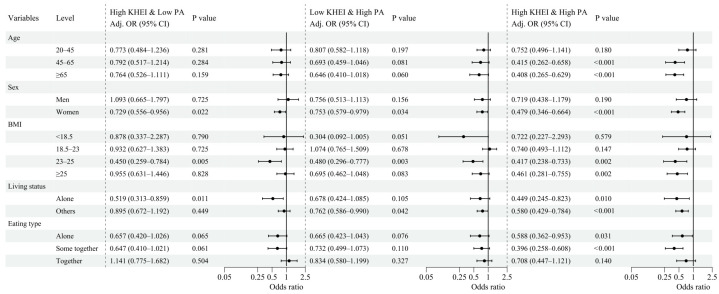
Subgroup Analyses of the Associations Between Lifestyle Groups and Depressive Symptoms. Survey-weighted fully adjusted odds ratios (aORs) and 95% confidence intervals for depressive symptoms across lifestyle groups within subgroups defined by sex, age, BMI, living status, and eating type. Models were adjusted for household income, educational level, marital status, smoking status, alcohol consumption, CCVD, cancer, and medication use for hypertension, DM, or dyslipidemia. The Low KHEI & Low PA group served as the reference category.

Subgroup analyses demonstrated variation in the associations between lifestyle groups and depressive symptoms across sex, age, BMI categories, living status, and eating type (Figure 3).

In sex-stratified analyses, none of the lifestyle groups were statistically significant among men. In contrast, among women, both the High KHEI & Low PA (aOR 0.729; 95% CI, 0.556–0.956) and Low KHEI & High PA (aOR 0.753; 95% CI, 0.579–0.979) groups showed significantly lower odds of depressive symptoms, with the strongest association observed in the High KHEI & High PA group (aOR 0.479; 95% CI, 0.346–0.664).

Age-stratified analyses showed distinct patterns. Among adults aged 20–45 years, no lifestyle group showed a statistically significant association with depressive symptoms. In those aged 45–65 years, only the High KHEI & High PA group (aOR 0.415; 95% CI, 0.262–0.658) was significant. Among adults aged ≥65 years, similarly, only the High KHEI & High PA group remained significant (aOR 0.408; 95% CI, 0.265–0.629).

In BMI-stratified analyses, no lifestyle group was associated with depressive symptoms among individuals with BMI < 18.5 kg/m^2^ or BMI 18.5–23 kg/m^2^. Among those with BMI 23–25 kg/m^2^, both the High KHEI & Low PA (aOR 0.450; 95% CI, 0.259–0.784) and High KHEI & High PA groups (aOR 0.417; 95% CI, 0.238–0.733) were significant. Among individuals with BMI ≥ 25 kg/m^2^, the High KHEI & High PA group showed a significant association (aOR 0.461; 95% CI, 0.281–0.755).

Living-status results also varied. Among individuals living alone, both the High KHEI & Low PA (aOR 0.519; 95% CI, 0.313–0.859) and High KHEI & High PA groups (aOR 0.449; 95% CI, 0.245–0.823) were significant. Among those living with others, only the High KHEI & High PA group remained significant (aOR 0.580; 95% CI, 0.429–0.784).

Across eating types, the High KHEI & High PA group showed significant associations with lower odds of depressive symptoms among individuals eating alone (aOR 0.588; 95% CI, 0.362–0.953) and those eating partially together (aOR 0.396; 95% CI, 0.258–0.608), but not among those eating together. No other lifestyle group reached statistical significance across eating type subgroups.

## 4. Discussion

### 4.1. Overview

In this nationally representative study of Korean adults, we identified joint associations between diet quality, physical activity, and depressive symptoms. Individuals with both low KHEI and low physical activity exhibited the highest likelihood of depressive symptoms, whereas those maintaining high levels of both behaviors demonstrated substantially lower odds. These findings suggest that the joint association of diet quality and physical activity with depressive symptoms may be more pronounced than their independent effects. Notably, meaningful variation was observed across sex and age groups, indicating that the associations of diet quality and physical activity with depressive symptoms vary across demographic contexts. This extends existing Western evidence to an Asian population [7], highlighting the importance of context-specific approaches.

### 4.2. Sex-Specific Patterns

Sex-specific differences were evident in our stratified analyses. Among women, all three lifestyle groups—High KHEI & Low PA, Low KHEI & High PA, and High KHEI & High PA—showed significantly lower odds of depressive symptoms compared with the Low KHEI & Low PA group, with the strongest association observed in the High KHEI & High PA group. In contrast, none of the lifestyle groups reached statistical significance among men. Supplementary Table S1 further supports this pattern: women with depressive symptoms had markedly lower scores across several key components of the KHEI—including breakfast consumption, fruit and vegetable intake, and protein-rich foods—compared with women without depressive symptoms, and they were also more likely to eat alone. Men showed similar directional differences, but the magnitude of these patterns was smaller and did not translate into significant lifestyle–depression associations in the male subgroup. These descriptive findings are consistent with Korean research suggesting that women may be more sensitive to the psychological implications of dietary habits and nutritional balance [25,26]. In East Asian cultural contexts, where communal eating carries strong social and gendered meaning, deviations such as solitary eating or poorer dietary adequacy may be associated with greater emotional impact among women [27]. Such behavioral and cultural factors may help explain why significant joint associations were observed in women but not in men.

### 4.3. Age-Specific Patterns

Age-stratified analyses revealed meaningful life-course variation in the associations between lifestyle factors and depressive symptoms. Among younger adults aged 20–45 years, no lifestyle group showed a statistically significant association with depressive symptoms. Supplementary Table S2 shows that younger adults with depressive symptoms had the lowest overall KHEI scores, particularly for breakfast, fruits, and vegetables, indicating that dietary inadequacy is most prominent in early adulthood. The absence of significant joint associations in this age group suggests that poor diet quality alone may not fully account for depressive symptoms in younger individuals. Prior Korean studies have shown that meal skipping and unstable eating routines are especially common in young adults and may be associated with a stronger influence on mental health than dietary composition itself [28,29]. This pattern implies that lifestyle instability—rather than solely the nutritional quality of the diet—may play a more central role in shaping depressive symptoms in early adulthood. In contrast, among adults aged 45–65 years and ≥65 years, only the High KHEI & High PA group showed significantly lower odds of depressive symptoms, suggesting that the simultaneous maintenance of both high diet quality and high physical activity may be particularly relevant for mental health in middle-aged and older adults. This finding underscores the particularly important role of physical activity in later life, consistent with evidence linking mobility and muscle health to psychological well-being in older adults [30,31]. Although our study did not directly assess the causal impact of solitary eating, its higher prevalence among older adults with depressive symptoms suggests that social context may further compound vulnerability to poor mental health in this population [32,33].

### 4.4. Additional Findings

Although sex and age accounted for much of the observed variation, several additional subgroup patterns offered further nuance. Among individuals with normal-to-overweight BMI ranges (23–25 and ≥25 kg/m^2^), the High KHEI & High PA group was consistently associated with lower odds of depressive symptoms, whereas no significant associations were observed among underweight or normal-weight individuals (BMI < 23 kg/m^2^). The lack of significant associations in the BMI < 18.5 subgroup may partly reflect impaired nutrient absorption in underweight individuals, where compromised gastrointestinal absorptive capacity can limit micronutrient bioavailability regardless of dietary quality [34], potentially attenuating the neurobiological benefits of a higher-quality diet through reduced tryptophan availability and serotonin synthesis [35]. Beyond BMI-related patterns, social context also appeared relevant: depressive symptoms were more prevalent among participants who lived or ate alone, yet even within these socially vulnerable groups, the combination of high KHEI and high physical activity was associated with lower odds of depressive symptoms. In contrast, no significant association was observed among those eating together, which may reflect the buffering role of social dining on mental health. These findings suggest that while social isolation and nutritional vulnerability are associated with elevated psychological risk, a combination of high diet quality and physical activity was linked to more favorable mental health profiles across diverse subgroups [6,36].

Beyond these subgroup patterns, examination of individual KHEI components revealed that the saturated fatty acid and sweets-and-beverage components did not differ significantly between participants with and without depressive symptoms. This pattern aligns with findings from Western HEI-based studies, in which saturated fat and added sugar components similarly showed no significant differences by depression status [37], and in which sugar-sweetened beverage intake alone was not independently associated with depressive symptoms [38]. Rather than fat or sugar moderation, adherence to traditional Korean meal structure—characterized by regular breakfast, adequate vegetable and protein intake, and sufficient energy—appears to be the more salient dietary dimension associated with depressive symptoms in this population. This divergence likely reflects the structural distinctiveness of the KHEI, which emphasizes traditional Korean dietary patterns centered on carbohydrate and vegetable adequacy, as distinct from the fat- and sugar-restriction focus of Western dietary indices.

### 4.5. Public Health Implications

From a public health perspective, these findings highlight the importance of integrated lifestyle approaches addressing diet quality and physical activity simultaneously. The observed pattern suggesting that the High KHEI & High PA group consistently showed the lowest odds of depressive symptoms across subgroups implies that targeting both behaviors concurrently may be more beneficial than focusing on a single factor. Given the variation across sex, age, and social circumstances, interventions should be tailored rather than uniform. Women and older adults—who showed stronger associations—may benefit most from combined nutritional and physical activity programs. Younger adults, who exhibited common dietary inadequacy but no significant joint associations, may require interventions stabilizing eating routines and reducing meal skipping. Although individuals living or eating alone showed higher prevalence of depressive symptoms, those with high diet quality and physical activity still demonstrated more favorable outcomes, suggesting multicomponent interventions may partially offset social isolation vulnerabilities. These results support culturally informed, multi-behavioral, subgroup-specific strategies to promote mental health.

### 4.6. Strengths and Limitations

This study has notable strengths, including its large, nationally representative sample and the use of validated instruments to assess both diet quality (KHEI) and depressive symptoms (PHQ-9). The application of complex survey weights accounting for the multistage stratified cluster sampling design of KNHANES further enhances the representativeness of the findings. The analyses incorporated extensive sociodemographic, behavioral, and clinical covariates, and the inclusion of detailed subgroup evaluations allowed for a more nuanced understanding of lifestyle–mental health relationships across diverse population groups.

Several limitations should also be acknowledged. The cross-sectional design precludes causal inference, and the possibility of reverse causality warrants careful consideration. Specifically, depressive symptoms may themselves lead to reduced physical activity and poorer dietary choices, rather than the reverse; individuals experiencing depression may be less motivated to engage in exercise or maintain a healthy diet, which could inflate the observed associations. While the exclusion of individuals with prevalent depression was intended to partially mitigate this concern by removing those with established diagnoses and associated treatment effects, residual reverse causality from subclinical depressive states cannot be fully excluded; furthermore, this exclusion may limit the generalizability of findings to individuals with active depression diagnoses. Longitudinal studies are needed to clarify the directionality of these associations. Longitudinal studies are needed to clarify the directionality of these associations. Dietary intake and physical activity were self-reported, introducing potential recall and reporting biases, and the KHEI does not fully capture eating behaviors such as snacking or meal timing, which may be particularly relevant for depressive symptoms. Residual confounding from unmeasured psychosocial factors may persist. Furthermore, the dichotomization of KHEI and physical activity at the median, while commonly used, may result in some loss of information; sensitivity analyses using continuous variables and quartiles yielded consistent results (Supplementary Table S3). Given that the median PA value in this sample was relatively low, an additional sensitivity analysis redefining high PA as the highest quartile was conducted; associations among men remained non-significant regardless of cutoff, suggesting sex-specific patterns rather than a threshold artifact (Supplementary Table S5). The subgroup analyses conducted across multiple demographic categories increase the risk of chance findings due to multiple comparisons; as no correction for multiple testing was applied, results from subgroup analyses should be interpreted with caution and regarded as exploratory rather than confirmatory. The BMI < 18.5 subgroup also showed wide confidence intervals owing to the small number of participants in this category (*n* = 673, 3.8%), which limits the interpretability of findings in this group; however, a sensitivity analysis excluding this subgroup confirmed that the overall results were not materially affected (Supplementary Table S4). Finally, depressive symptoms were identified using a screening instrument rather than clinical diagnoses, although the PHQ-9 cutoff applied in this study has demonstrated strong validity in Korean populations.

## 5. Conclusions

In conclusion, low diet quality and low physical activity were jointly associated with a higher likelihood of depressive symptoms in Korean adults, while the combination of high diet quality and high physical activity was associated with the lowest odds. These associations were particularly pronounced among women and middle-aged to older adults, but absent among younger adults and men. These findings highlight the need to account for demographic and cultural contexts—especially eating practices—when designing mental health–related lifestyle interventions and underscore the value of establishing population-specific evidence. Future longitudinal research is warranted to clarify causal pathways and to determine whether integrated, culturally tailored approaches that address both nutritional quality and physical activity can effectively reduce depressive symptoms across population subgroups.

## Figures and Tables

**Table 1:** Baseline Characteristics of Participants Across Lifestyle Groups.

Variables		Overall	Low KHEI & Low PA	High KHEI & Low PA	Low KHEI & High PA	High KHEI & High PA	*p* Value for Trend
*n*		17,737	4250	3897	4511	5079	
Age	Mean (SD)	51.58 (16.53)	50.60 (17.45)	56.32 (15.19)	45.41 (16.42)	54.26 (14.96)	<0.001
	20–45	6485 (36.6)	1770 (41.6)	984 (25.3)	2349 (52.1)	1382 (27.2)	<0.001
	45–65	6663 (37.6)	1414 (33.3)	1571 (40.3)	1465 (32.5)	2213 (43.6)	
	≥65	4589 (25.9)	1066 (25.1)	1342 (34.4)	697 (15.5)	1484 (29.2)	
Sex	Men	7488 (42.2)	1736 (40.8)	1266 (32.5)	2273 (50.4)	2213 (43.6)	<0.001
	Women	10,249 (57.8)	2514 (59.2)	2631 (67.5)	2238 (49.6)	2866 (56.4)	
BMI	Mean (SD)	23.94 (3.53)	23.60 (3.66)	23.64 (3.44)	24.41 (3.80)	24.04 (3.19)	<0.001
	<18.5	673 (3.8)	245 (5.8)	178 (4.6)	137 (3.0)	113 (2.2)	<0.001
	18.5–23	6833 (38.5)	1736 (40.8)	1627 (41.8)	1582 (35.1)	1888 (37.2)	
	23–25	4066 (22.9)	901 (21.2)	882 (22.6)	1005 (22.3)	1278 (25.2)	
	≥25	6165 (34.8)	1368 (32.2)	1210 (31.0)	1787 (39.6)	1800 (35.4)	
Household income	Low	7503 (42.3)	2080 (48.9)	1814 (46.5)	1703 (37.8)	1906 (37.5)	<0.001
	Middle	4996 (28.2)	1141 (26.8)	1050 (26.9)	1370 (30.4)	1435 (28.3)	
	High	5238 (29.5)	1029 (24.2)	1033 (26.5)	1438 (31.9)	1738 (34.2)	
Educational level	Less than high school	5413 (30.5)	1498 (35.2)	1570 (40.3)	943 (20.9)	1402 (27.6)	<0.001
	High school graduate	5725 (32.3)	1349 (31.7)	1146 (29.4)	1587 (35.2)	1643 (32.3)	
	Beyond high school	6599 (37.2)	1403 (33.0)	1181 (30.3)	1981 (43.9)	2034 (40.0)	
Marital status	Married	14,978 (84.4)	3528 (83.0)	3637 (93.3)	3285 (72.8)	4528 (89.2)	<0.001
	Unmarried	2759 (15.6)	722 (17.0)	260 (6.7)	1226 (27.2)	551 (10.8)	
Living status	Others	15,668 (88.3)	3635 (85.5)	3425 (87.9)	3998 (88.6)	4610 (90.8)	<0.001
	Alone	2069 (11.7)	615 (14.5)	472 (12.1)	513 (11.4)	469 (9.2)	
Eating type	Together	8294 (46.8)	2029 (47.7)	1803 (46.3)	2150 (47.7)	2312 (45.5)	<0.001
	Some together	7155 (40.3)	1539 (36.2)	1602 (41.1)	1766 (39.1)	2248 (44.3)	
	Alone	2288 (12.9)	682 (16.0)	492 (12.6)	595 (13.2)	519 (10.2)	
Smoking status	Never	10,949 (61.7)	2438 (57.4)	2734 (70.2)	2509 (55.6)	3268 (64.3)	<0.001
	Former	3803 (21.4)	838 (19.7)	682 (17.5)	1034 (22.9)	1249 (24.6)	
	Current	2985 (16.8)	974 (22.9)	481 (12.3)	968 (21.5)	562 (11.1)	
Alcohol consumption	Non-drinker	8429 (47.5)	2027 (47.7)	2230 (57.2)	1719 (38.1)	2453 (48.3)	<0.001
	Non-high	7424 (41.9)	1697 (39.9)	1411 (36.2)	2120 (47.0)	2196 (43.2)	
	High-risk	1884 (10.6)	526 (12.4)	256 (6.6)	672 (14.9)	430 (8.5)	
CCVD		883 (5.0)	246 (5.8)	217 (5.6)	148 (3.3)	272 (5.4)	<0.001
Cancer		944 (5.3)	189 (4.4)	217 (5.6)	187 (4.1)	351 (6.9)	<0.001
Hypertension		3949 (22.3)	923 (21.7)	1090 (28.0)	718 (15.9)	1218 (24.0)	<0.001
Diabetes mellitus		1612 (9.1)	372 (8.8)	462 (11.9)	296 (6.6)	482 (9.5)	<0.001
Dyslipidemia		2258 (12.7)	440 (10.4)	623 (16.0)	419 (9.3)	776 (15.3)	<0.001

Baseline characteristics of 17,737 adults according to four lifestyle groups defined by diet quality (KHEI) and physical activity (PA): Low KHEI & Low PA, High KHEI & Low PA, Low KHEI & High PA, and High KHEI & High PA. Values are presented as number (%) for categorical variables and mean (SD) for continuous variables. *p* values were calculated using chi-square tests for categorical variables and ANOVA for continuous variables.

**Table 2:** Baseline Characteristics of Participants With and Without Depressive Symptoms.

Variables		Without Depressive Symptoms		With Depressive Symptoms			*p* Value for Trend
		Minimal	Mild	Moderate	Moderately Severe	Severe	
*n*		14,530	2385	570	179	73	
KHEI	Mean (SD)	63.93 (13.18)	61.64 (13.44)	60.27 (13.94)	57.80 (13.66)	54.82 (13.62)	<0.001
	Have breakfast (0–10)	7.65 (3.67)	6.84 (4.02)	6.62 (4.17)	7.03 (4.08)	6.55 (4.42)	<0.001
	Mixed grain intake (0–5)	2.18 (2.17)	1.95 (2.15)	2.07 (2.16)	1.92 (2.17)	1.67 (2.16)	<0.001
	Total fruit intake (0–5)	2.38 (2.20)	2.26 (2.21)	2.00 (2.18)	1.88 (2.19)	2.03 (2.24)	<0.001
	Fresh fruit intake (0–5)	2.56 (2.38)	2.44 (2.39)	2.19 (2.38)	2.02 (2.37)	2.18 (2.41)	<0.001
	Total vegetables intake (0–5)	3.57 (1.45)	3.36 (1.54)	3.29 (1.61)	3.10 (1.61)	2.50 (1.47)	<0.001
	pickled vegetables intake (0–5)	3.31 (1.62)	3.09 (1.68)	3.07 (1.71)	2.75 (1.79)	2.32 (1.62)	<0.001
	Meat, fish, egg & beans intake (0–10)	7.13 (3.08)	6.95 (3.22)	6.56 (3.47)	5.42 (3.53)	4.91 (3.61)	<0.001
	Milk and milk products intake (0–10)	3.24 (4.38)	3.16 (4.33)	2.98 (4.24)	2.68 (4.15)	2.75 (4.17)	0.204
	Percentage of energy from saturated fatty acid (0–10)	7.63 (3.82)	7.46 (3.92)	7.67 (3.90)	7.58 (3.95)	7.19 (4.15)	0.288
	Sodium intake (0–10)	6.76 (3.28)	7.06 (3.19)	7.26 (3.25)	7.64 (3.07)	7.97 (2.91)	<0.001
	Percentage of energy from sweets and beverage (0–10)	6.64 (3.78)	6.41 (3.95)	6.50 (3.78)	5.90 (4.19)	5.32 (4.27)	0.379
	Percentage of energy from carbohydrates (0–5)	2.44 (2.11)	2.35 (2.16)	2.33 (2.15)	1.96 (2.10)	1.81 (2.14)	0.001
	Percentage of energy from fat (0–5)	3.29 (2.13)	3.19 (2.16)	3.14 (2.20)	2.65 (2.30)	2.84 (2.32)	<0.001
	Energy intake (0–5)	3.14 (2.21)	3.03 (2.23)	2.68 (2.31)	2.65 (2.31)	2.13 (2.28)	<0.001
Physical activity		188.39 (252.46)	154.99 (212.99)	145.29 (231.64)	150.92 (249.96)	130.52 (241.49)	<0.001
Lifestyle	Low KHEI & Low PA	3283 (22.6)	670 (28.1)	193 (33.9)	67 (37.4)	37 (50.7)	<0.001
	High KHEI & Low PA	3169 (21.8)	555 (23.3)	129 (22.6)	34 (19.0)	10 (13.7)	
	Low KHEI & High PA	3665 (25.2)	631 (26.5)	147 (25.8)	49 (27.4)	19 (26.0)	
	High KHEI & High PA	4413 (30.4)	529 (22.2)	101 (17.7)	29 (16.2)	7 (9.6)	
Age	Mean (SD)	51.88 (16.24)	49.38 (17.35)	51.33 (18.47)	56.17 (17.94)	55.18 (19.60)	<0.001
	20–45	5142 (35.4)	1039 (43.6)	227 (39.8)	51 (28.5)	26 (35.6)	<0.001
	45–65	5653 (38.9)	773 (32.4)	170 (29.8)	55 (30.7)	12 (16.4)	
	≥65	3735 (25.7)	573 (24.0)	173 (30.4)	73 (40.8)	35 (47.9)	
Sex	Men	6481 (44.6)	768 (32.2)	162 (28.4)	59 (33.0)	18 (24.7)	<0.001
	Women	8049 (55.4)	1617 (67.8)	408 (71.6)	120 (67.0)	55 (75.3)	
BMI	Mean (SD)	23.99 (3.47)	23.66 (3.78)	23.92 (3.88)	24.08 (3.78)	23.25 (3.83)	<0.001
	<18.5	482 (3.3)	142 (6.0)	33 (5.8)	9 (5.0)	7 (9.6)	<0.001
	18.5–23	5499 (37.8)	1014 (42.5)	223 (39.1)	65 (36.3)	32 (43.8)	
	23–25	3439 (23.7)	465 (19.5)	116 (20.4)	36 (20.1)	10 (13.7)	
	≥25	5110 (35.2)	764 (32.0)	198 (34.7)	69 (38.5)	24 (32.9)	
Household income	Low	5884 (40.5)	1117 (46.8)	317 (55.6)	135 (75.4)	50 (68.5)	<0.001
	Middle	4161 (28.6)	640 (26.8)	151 (26.5)	28 (15.6)	16 (21.9)	
	High	4485 (30.9)	628 (26.3)	102 (17.9)	16 (8.9)	7 (9.6)	
Educational level	Less than high school	4295 (29.6)	752 (31.5)	230 (40.4)	99 (55.3)	37 (50.7)	<0.001
	High school graduate	4697 (32.3)	786 (33.0)	176 (30.9)	41 (22.9)	25 (34.2)	
	Beyond high school	5538 (38.1)	847 (35.5)	164 (28.8)	39 (21.8)	11 (15.1)	
Marital status	Married	12,438 (85.6)	1880 (78.8)	458 (80.4)	143 (79.9)	59 (80.8)	<0.001
	Unmarried	2092 (14.4)	505 (21.2)	112 (19.6)	36 (20.1)	14 (19.2)	
Living status	Others	12,996 (89.4)	2036 (85.4)	455 (79.8)	126 (70.4)	55 (75.3)	<0.001
	Alone	1534 (10.6)	349 (14.6)	115 (20.2)	53 (29.6)	18 (24.7)	
Eating type	Together	6981 (48.0)	1001 (42.0)	234 (41.1)	53 (29.6)	25 (34.2)	<0.001
	Some together	5910 (40.7)	995 (41.7)	179 (31.4)	55 (30.7)	16 (21.9)	
	Alone	1639 (11.3)	389 (16.3)	157 (27.5)	71 (39.7)	32 (43.8)	
Smoking status	Never	8942 (61.5)	1530 (64.2)	333 (58.4)	106 (59.2)	38 (52.1)	<0.001
	Former	3263 (22.5)	395 (16.6)	99 (17.4)	34 (19.0)	12 (16.4)	
	Current	2325 (16.0)	460 (19.3)	138 (24.2)	39 (21.8)	23 (31.5)	
Alcohol consumption	Non-drinker	6879 (47.3)	1128 (47.3)	289 (50.7)	97 (54.2)	36 (49.3)	0.001
	Non-high	6154 (42.4)	983 (41.2)	208 (36.5)	53 (29.6)	26 (35.6)	
	High-risk	1497 (10.3)	274 (11.5)	73 (12.8)	29 (16.2)	11 (15.1)	
CCVD		673 (4.6)	124 (5.2)	49 (8.6)	24 (13.4)	13 (17.8)	<0.001
Cancer		743 (5.1)	144 (6.0)	39 (6.8)	15 (8.4)	3 (4.1)	0.043
Hypertension		3243 (22.3)	494 (20.7)	143 (25.1)	51 (28.5)	18 (24.7)	0.037
Diabetes mellitus		1265 (8.7)	228 (9.6)	81 (14.2)	24 (13.4)	14 (19.2)	<0.001
Dyslipidemia		1823 (12.5)	302 (12.7)	92 (16.1)	27 (15.1)	14 (19.2)	0.040

Baseline characteristics of 17,737 adults stratified by depressive symptoms status: Minimal depressive symptoms (PHQ-9 0–4), Mild (PHQ-9 5–9), Moderate (PHQ-9 10–14), Moderately severe (PHQ-9 15–19), and Severe (PHQ-9 20–27). The presence of depressive symptoms was defined as a PHQ-9 score ≥ 10. Values are presented as number (%) for categorical variables and mean (SD) for continuous variables. *p* values were calculated using chi-square tests for categorical variables and ANOVA tests for continuous variables.

## Data Availability

The datasets analyzed during the current study are publicly available from the Korea National Health and Nutrition Examination Survey website (https://knhanes.kdca.go.kr, accessed on 11 March 2026) upon reasonable request and approval by the Korea Disease Control and Prevention Agency [39]. (Accessed on 3 April 2025).

## Supplementary Materials

The following supporting information can be downloaded at https://www.mdpi.com/article/10.3390/nu18060915/s1: Table S1: Sex-stratified KHEI and Eating Type by Depressive Status; Table S2: Age-stratified KHEI and Eating Type by Depressive Status; Table S3: Sensitivity Analyses Using KHEI and Physical Activity as Continuous Variables and Quartiles; Table S4: Sensitivity Analysis Excluding Underweight Individuals (BMI < 18.5 kg/m^2^); Table S5: Sensitivity Analysis Using the Highest Physical Activity Quartile (Q4) as the High PA Threshold.

## Abbreviations

The following abbreviations are used in this manuscript:

KHEI	Korean Healthy Eating Index
PA	Physical Activity
BMI	Body Mass Index
CCVD	Cardiovascular or Cerebrovascular Disease
DM	Diabetes Mellitus
OR	Odds Ratio
aOR	Adjusted Odds Ratio
CI	Confidence Interval
FFQ	Food Frequency Questionnaire
MET	Metabolic Equivalent of Task
PHQ-9	Patient Health Questionnaire-9
DSM-IV	Diagnostic and Statistical Manual of Mental Disorders, Fourth Edition
IRB	Institutional Review Board
KNHANES	Korea National Health and Nutrition Examination Survey
