# Supplementary material for: Sex- and Age-Specific Differences in the Joint Effects of Diet Quality and Physical Activity on Depressive Symptoms in Adults: A Cross-Sectional Study
Source: Nutrients. 2026 Mar 13;18(6):915. doi: 10.3390/nu18060915 (PMC13028771; doi:10.3390/nu18060915)
Supplement: Supplementary file 1 [file nutrients-18-00915-s001.zip › nutrients-4178617-supplementary.pdf]

Table S1. Sex-stratified KHEI and Eating Type by Depressive Status

Variables		Men		P value	Women		P value
		With depressive symptoms	Without depressive symptoms		With depressive symptoms	Without depressive symptoms	
n		239	7249		583	9666	
KHEI	Mean (SD)	57.77 (13.72)	62.03 (12.94)	<0.001	59.86 (13.99)	64.79 (13.34)	<0.001
	Item 1	6.71 (4.18)	7.54 (3.76)	0.001	6.70 (4.17)	7.53 (3.71)	<0.001
	Item 2	1.98 (2.24)	2.15 (2.20)	0.226	2.01 (2.13)	2.15 (2.14)	0.149
	Item 3	1.50 (2.04)	1.89 (2.10)	0.004	2.17 (2.21)	2.71 (2.21)	<0.001
	Item 4	1.61 (2.27)	2.09 (2.33)	0.002	2.37 (2.39)	2.87 (2.36)	<0.001
	Item 5	3.40 (1.59)	3.81 (1.38)	<0.001	3.09 (1.61)	3.34 (1.49)	<0.001
	Item 6	2.99 (1.71)	3.39 (1.61)	<0.001	2.91 (1.75)	3.19 (1.64)	<0.001
	Item 7	6.45 (3.45)	7.34 (3.02)	<0.001	6.04 (3.58)	6.93 (3.16)	<0.001
	Item 8	2.62 (4.16)	2.87 (4.27)	0.362	3.01 (4.23)	3.49 (4.43)	0.010
	Item 9	7.34 (4.01)	7.57 (3.86)	0.359	7.72 (3.89)	7.63 (3.82)	0.593
	Item 10	6.32 (3.46)	5.69 (3.44)	0.005	7.85 (2.97)	7.63 (2.86)	0.072
	Item 11	6.62 (3.81)	7.20 (3.56)	0.254	6.14 (3.93)	6.13 (3.92)	0.980

	Item 12	2.30 (2.13)	2.50 (2.12)	0.162	2.16 (2.15)	2.37 (2.12)	0.021
	Item 13	3.16 (2.23)	3.31 (2.12)	0.272	2.95 (2.25)	3.24 (2.14)	0.001
	Item 14	2.87 (2.30)	3.14 (2.23)	0.069	2.53 (2.31)	3.11 (2.21)	<0.001
Eating type	Together	104 (43.5)	3866 (53.3)	<0.001	208 (35.7)	4116 (42.6)	<0.001
	Some together	55 (23.0)	2660 (36.7)		195 (33.4)	4245 (43.9)	
	Alone	80 (33.5)	723 (10.0)		180 (30.9)	1305 (13.5)	

KHEI items were numbered 1–14. The scoring range for each item is indicated in parentheses. KHEI Item 1: breakfast consumption (0–10); Item 2: mixed grain intake (0–5); Item 3: total fruit intake (0–5); Item 4: fresh fruit intake (0–5); Item 5: total vegetable intake (0–5); Item 6: vegetable intake excluding Kimchi and pickled vegetables (0–5); Item 7: meat, fish, egg, and beans intake (0–10); Item 8: milk and dairy intake (0–10); Item 9: percentage of energy from saturated fatty acid (0–10); Item 10: sodium intake (0–10); Item 11: percentage of energy from sweets and beverages (0–10); Item 12: percentage of energy from carbohydrates (0–5); Item 13: percentage of energy from fat (0–5); Item 14: total energy intake (0–5).

Table S2. Age-stratified KHEI and Eating Type by Depressive Status

Variable		20-45 years		P value	45-65 years		P value	≥65 years		P value
		With depressive symptoms	Without depressive symptoms		With depressive symptoms	Without depressive symptoms		With depressive symptoms	Without depressive symptoms	
n		304	6181		237	6426		281	4308	
KHEI	Mean (SD)	54.26 (13.18)	58.91 (13.41)	<0.001	60.71 (13.80)	65.65 (12.56)	<0.001	63.42 (13.23)	67.31 (11.96)	<0.001
	Item 1	4.09 (4.03)	5.62 (4.06)	<0.001	7.16 (4.01)	8.02 (3.47)	<0.001	9.16 (2.50)	9.56 (1.86)	0.001
	Item 2	1.44 (2.01)	1.59 (2.03)	0.218	2.06 (2.11)	2.34 (2.17)	0.050	2.56 (2.22)	2.67 (2.19)	0.447
	Item 3	1.50 (1.95)	1.81 (2.07)	0.010	2.11 (2.21)	2.61 (2.17)	0.001	2.38 (2.31)	2.78 (2.28)	0.005
	Item 4	1.68 (2.21)	2.02 (2.31)	0.013	2.34 (2.40)	2.83 (2.34)	0.002	2.49 (2.46)	2.86 (2.40)	0.014
	Item 5	2.87 (1.59)	3.21 (1.51)	<0.001	3.32 (1.56)	3.74 (1.37)	<0.001	3.39 (1.63)	3.73 (1.43)	<0.001
	Item 6	2.80 (1.72)	2.99 (1.62)	0.044	3.01 (1.70)	3.43 (1.57)	<0.001	3.02 (1.78)	3.46 (1.66)	<0.001
	Item 7	7.10 (3.27)	7.50 (2.90)	0.020	5.90 (3.31)	7.01 (3.06)	<0.001	5.36 (3.79)	6.68 (3.37)	<0.001
	Item 8	3.37 (4.31)	3.68 (4.50)	0.252	2.89 (4.32)	3.25 (4.40)	0.219	2.38 (3.96)	2.55 (4.05)	0.489
	Item 9	6.05 (4.47)	6.21 (4.34)	0.533	7.85 (3.75)	7.99 (3.57)	0.561	9.09 (2.63)	9.04 (2.59)	0.752

	Item 10	6.60 (3.44)	6.44 (3.35)	0.429	7.29 (3.17)	6.62 (3.30)	0.002	8.38 (2.62)	7.60 (2.96)	<0.001
	Item 11	5.88 (4.05)	6.84 (3.76)	0.038	6.43 (3.88)	6.39 (3.81)	0.949	6.89 (3.60)	6.64 (3.84)	0.688
	Item 12	2.73 (2.12)	2.79 (2.11)	0.678	2.48 (2.09)	2.52 (2.09)	0.726	1.40 (1.97)	1.76 (2.02)	0.004
	Item 13	3.62 (2.00)	3.56 (2.03)	0.664	3.32 (2.16)	3.43 (2.06)	0.425	2.08 (2.27)	2.61 (2.25)	<0.001
	Item 14	2.52 (2.31)	3.02 (2.24)	<0.001	2.90 (2.27)	3.18 (2.21)	0.058	2.51 (2.34)	3.19 (2.19)	<0.001
Eating type	Together	132 (43.4)	3056 (49.4)	<0.001	81 (34.2)	2842 (44.2)	<0.001	99 (35.2)	2084 (48.4)	<0.001
	Some together	116 (38.2)	2689 (43.5)		86 (36.3)	2915 (45.4)		48 (17.1)	1301 (30.2)	
	Alone	56 (18.4)	436 (7.1)		70 (29.5)	669 (10.4)		134 (47.7)	923 (21.4)	

KHEI items were numbered 1–14. The scoring range for each item is indicated in parentheses. KHEI Item 1: breakfast consumption (0–10); Item 2: mixed grain intake (0–5); Item 3: total fruit intake (0–5); Item 4: fresh fruit intake (0–5); Item 5: total vegetable intake (0–5); Item 6: vegetable intake excluding Kimchi and pickled vegetables (0–5); Item 7: meat, fish, egg, and beans intake (0–10); Item 8: milk and dairy intake (0–10); Item 9: percentage of energy from saturated fatty acid (0–10); Item 10: sodium intake (0–10); Item 11: percentage of energy from sweets and beverages (0–10); Item 12: percentage of energy from carbohydrates (0–5); Item 13: percentage of energy from fat (0–5); Item 14: total energy intake (0–5).

Table S3. Sensitivity Analyses Using KHEI and Physical Activity as Continuous Variables and Quartiles

Model	Variables	Adjusted OR	95% CI	P value
Model 1	KHEI	0.987	0.980–0.994	<0.001
	PA	1.000	0.999–1.000	0.392
Model 2 (KHEI)	Q1 (Lowest) [Reference]	1		
	Q2	0.780	0.631–0.963	0.021
	Q3	0.718	0.557–0.927	0.011
	Q4 (Highest)	0.639	0.499–0.819	<0.001
Model 3 (PA)	Q1 (Lowest) [Reference]	1		
	Q2	0.719	0.572–0.904	0.005
	Q3	0.571	0.449–0.725	<0.001
	Q4 (Highest)	0.653	0.500–0.854	0.002

Adjusted odds ratios (aORs) and 95% confidence intervals (CIs) from survey-weighted logistic regression models examining the associations between diet quality (KHEI) and physical activity (PA) with depressive symptoms. Model 1 presents KHEI and PA as continuous variables. Models 2 and 3 present KHEI and PA categorized into quartiles, respectively, with Q1 (lowest) as the reference category. Models were adjusted for age, sex, BMI, household income, educational level, marital status, living status, eating type, smoking status, alcohol consumption, CCVD, cancer, and medication use for hypertension, DM, or dyslipidemia.

Table S4. Sensitivity Analysis Excluding Underweight Individuals (BMI <18.5 kg/m²)

Model	Group	aOR	95% CI	P value
Original (BMI <18.5 included)	Low KHEI & Low PA [Reference]	1		
Original (BMI <18.5 included)	High KHEI & Low PA	0.813	0.633–1.044	0.105
Original (BMI <18.5 included)	Low KHEI & High PA	0.744	0.593–0.933	0.011
Original (BMI <18.5 included)	High KHEI & High PA	0.553	0.421–0.726	<0.001
Sensitivity (BMI <18.5 excluded)	Low KHEI & Low PA [Reference]	1		
Sensitivity (BMI <18.5 excluded)	High KHEI & Low PA	0.820	0.637–1.056	0.124
Sensitivity (BMI <18.5 excluded)	Low KHEI & High PA	0.787	0.624–0.992	0.043
Sensitivity (BMI <18.5 excluded)	High KHEI & High PA	0.562	0.425–0.743	<0.001

Adjusted odds ratios (aORs) and 95% confidence intervals (CIs) from survey-weighted logistic regression models examining the associations between diet quality (KHEI) and physical activity (PA) with depressive symptoms. The reference group is Low KHEI & Low PA. Models were adjusted for age, sex, BMI, household income, educational level, marital status, living status, eating type, smoking status, alcohol consumption, CCVD, cancer, and medication use for hypertension, DM, or dyslipidemia.

Table S5. Sensitivity Analysis Using the Highest Physical Activity Quartile (Q4) as the High PA Threshold

Subgroup	Level	Group	OR	95% CI	P value
Sex	Men	Low KHEI & Low PA [Reference]	1.000		
		High KHEI & Low PA	0.914	0.603–1.384	0.669
		Low KHEI & High PA	0.885	0.581–1.349	0.569
		High KHEI & High PA	0.838	0.477–1.473	0.540
	Women	Low KHEI & Low PA [Reference]	1.000		
		High KHEI & Low PA	0.624	0.499–0.781	<.001
		Low KHEI & High PA	0.998	0.677–1.471	0.994
		High KHEI & High PA	0.492	0.313–0.774	0.002
Age	20–45	Low KHEI & Low PA [Reference]	1.000		
		High KHEI & Low PA	0.682	0.476–0.978	0.037
		Low KHEI & High PA	1.115	0.750–1.658	0.589
		High KHEI & High PA	1.035	0.628–1.706	0.892
	45–65	Low KHEI & Low PA [Reference]	1.000		
		High KHEI & Low PA	0.640	0.450–0.912	0.014
		Low KHEI & High PA	0.710	0.418–1.205	0.204

		High KHEI & High PA	0.315	0.170–0.587	<.001
	≥65	Low KHEI & Low PA [Reference]	1.000		
		High KHEI & Low PA	0.644	0.474–0.876	0.005
		Low KHEI & High PA	0.510	0.278–0.935	0.029
		High KHEI & High PA	0.340	0.192–0.605	<.001

Adjusted odds ratios (aORs) and 95% confidence intervals (CIs) from survey-weighted logistic regression models examining the associations between diet quality (KHEI) and physical activity (PA) with depressive symptoms, stratified by sex and age. In this sensitivity analysis, the high PA threshold was redefined as the highest quartile (Q4) of PA energy expenditure, in contrast to the median cutoff used in the primary analysis. The reference group is Low KHEI & Low PA within each subgroup. Models were adjusted for age (sex-stratified models only), sex (age-stratified models only), BMI, household income, educational level, marital status, living status, eating type, smoking status, alcohol consumption, CCVD, cancer, and medication use for hypertension, DM, or dyslipidemia.
